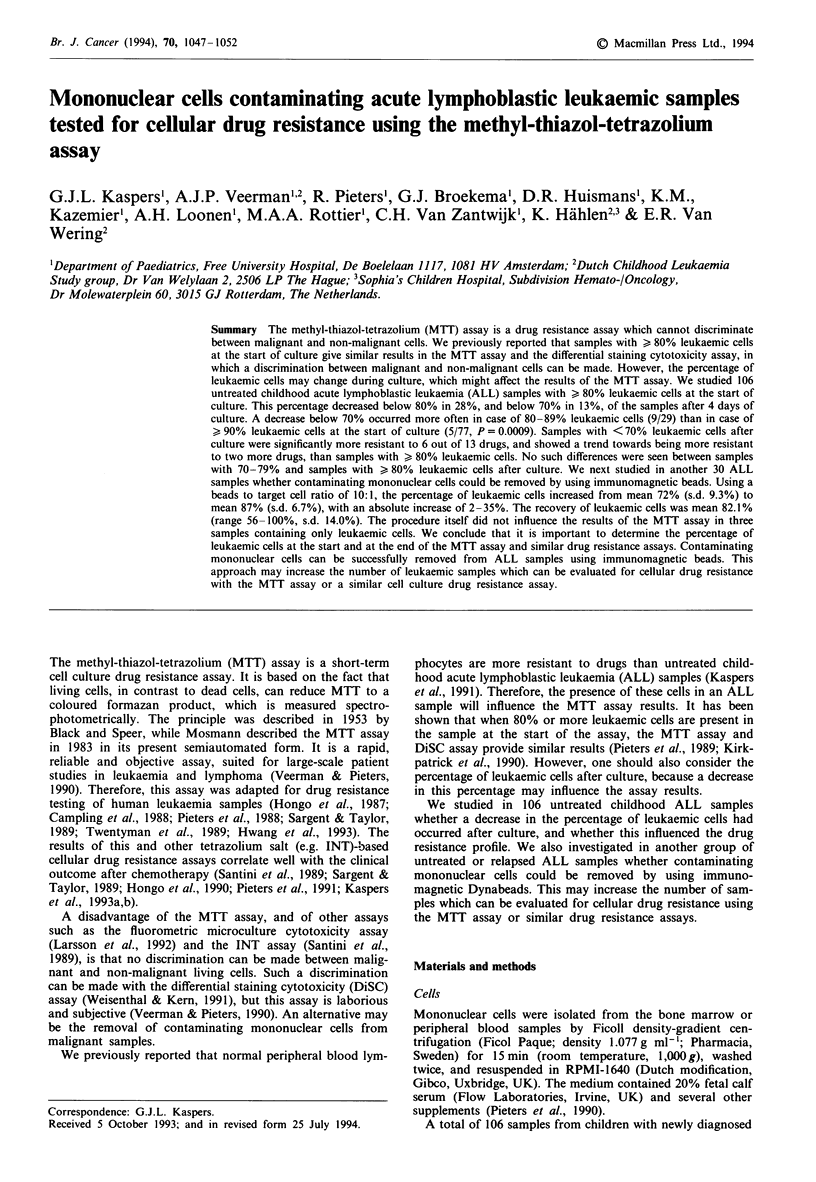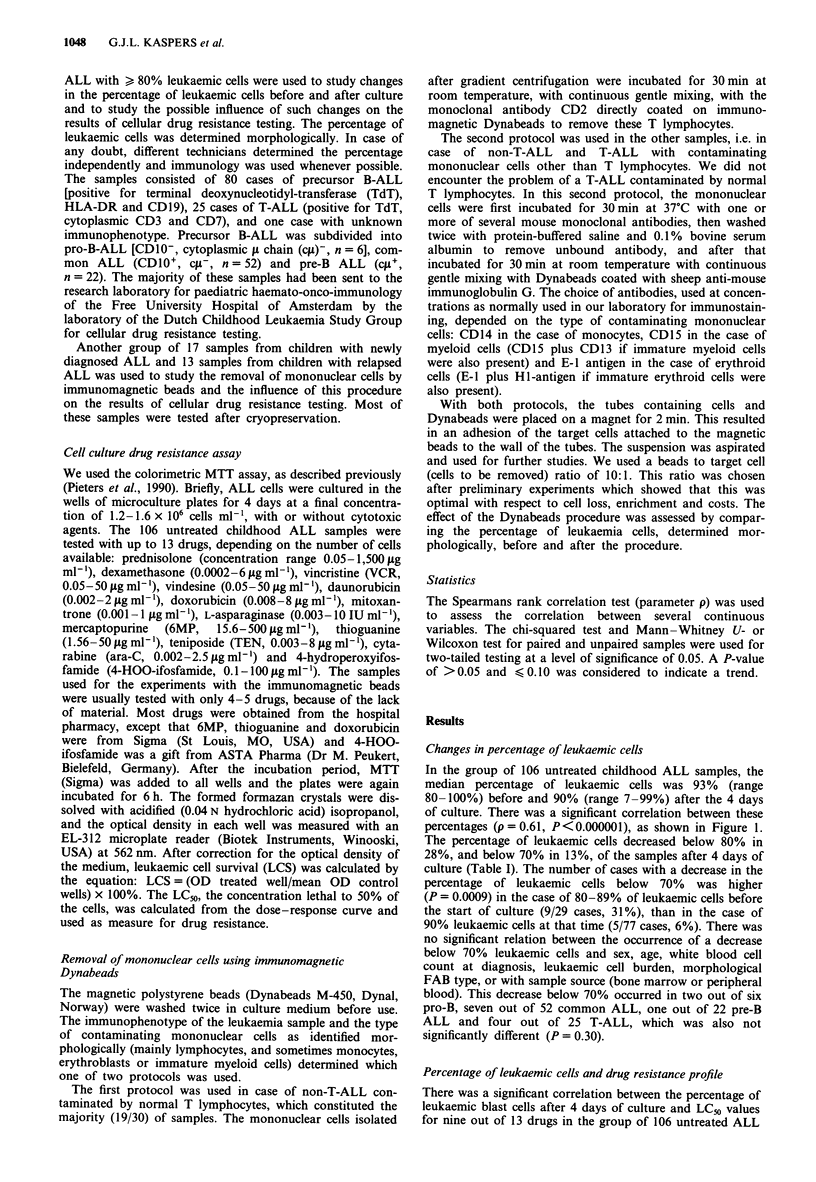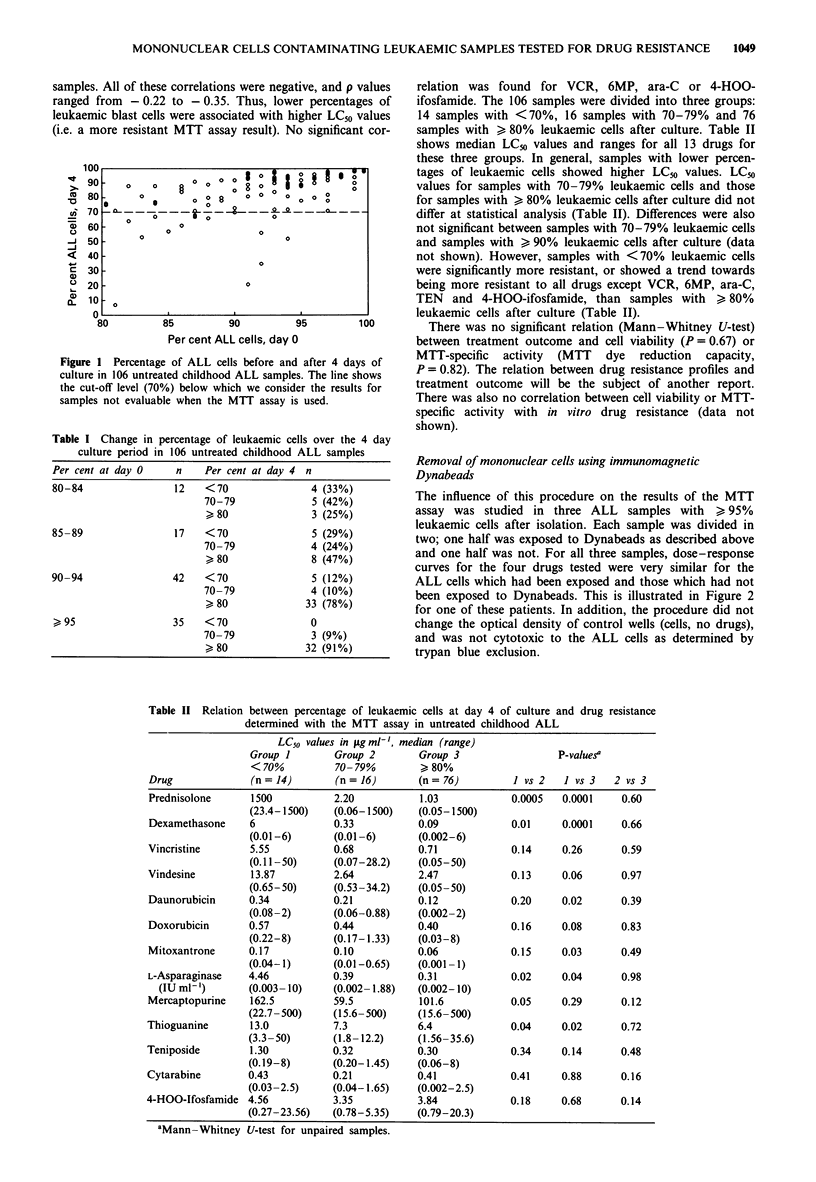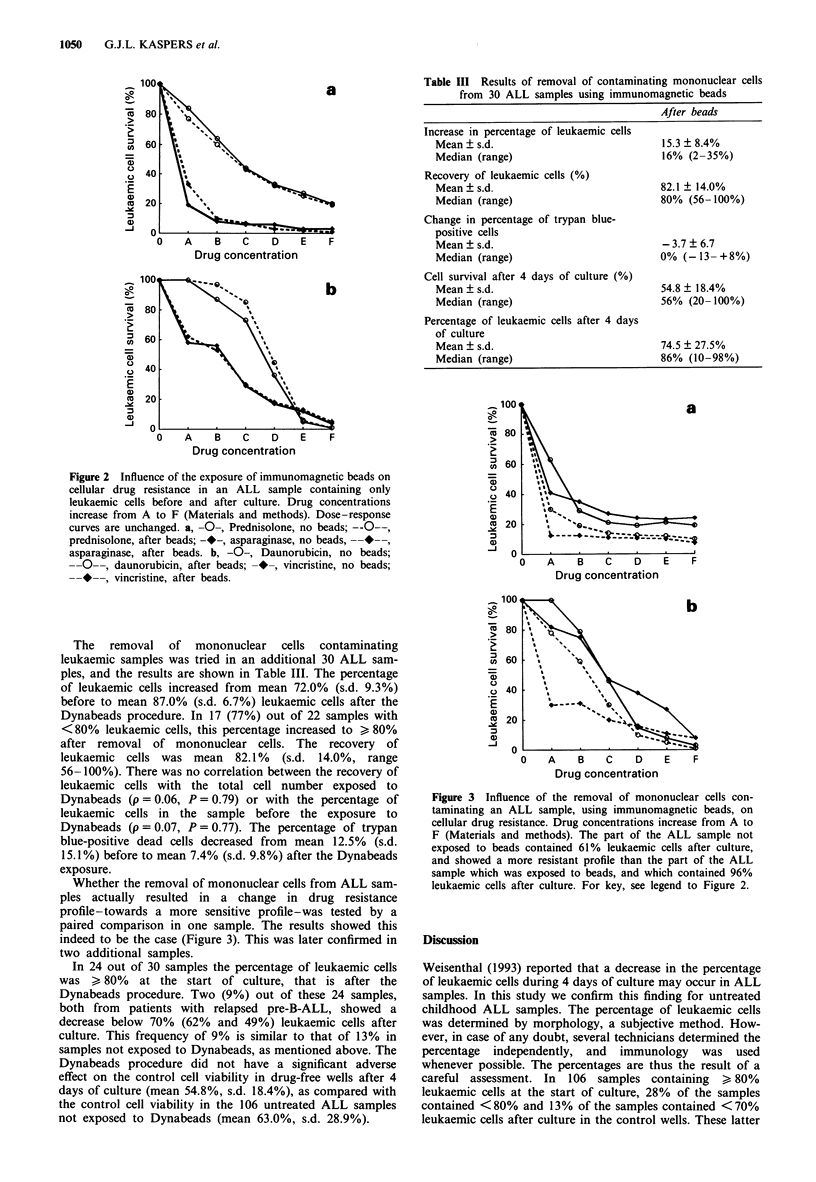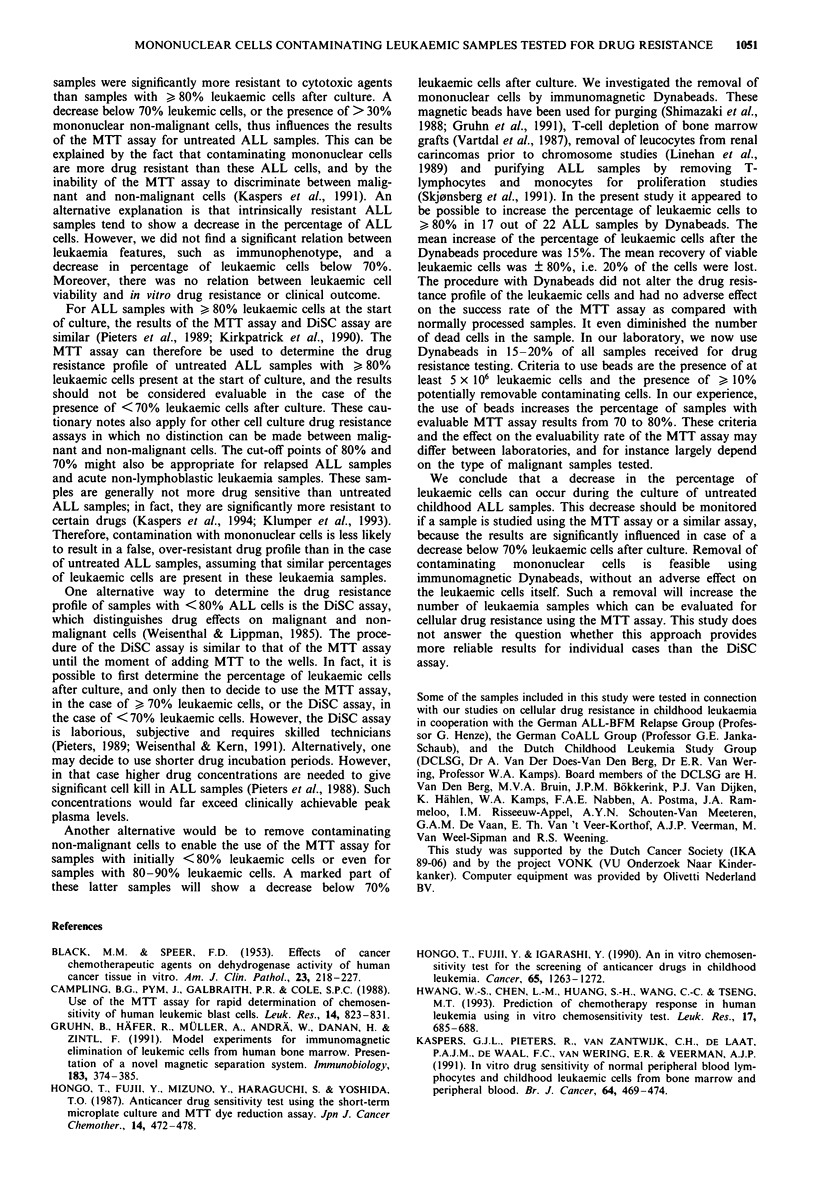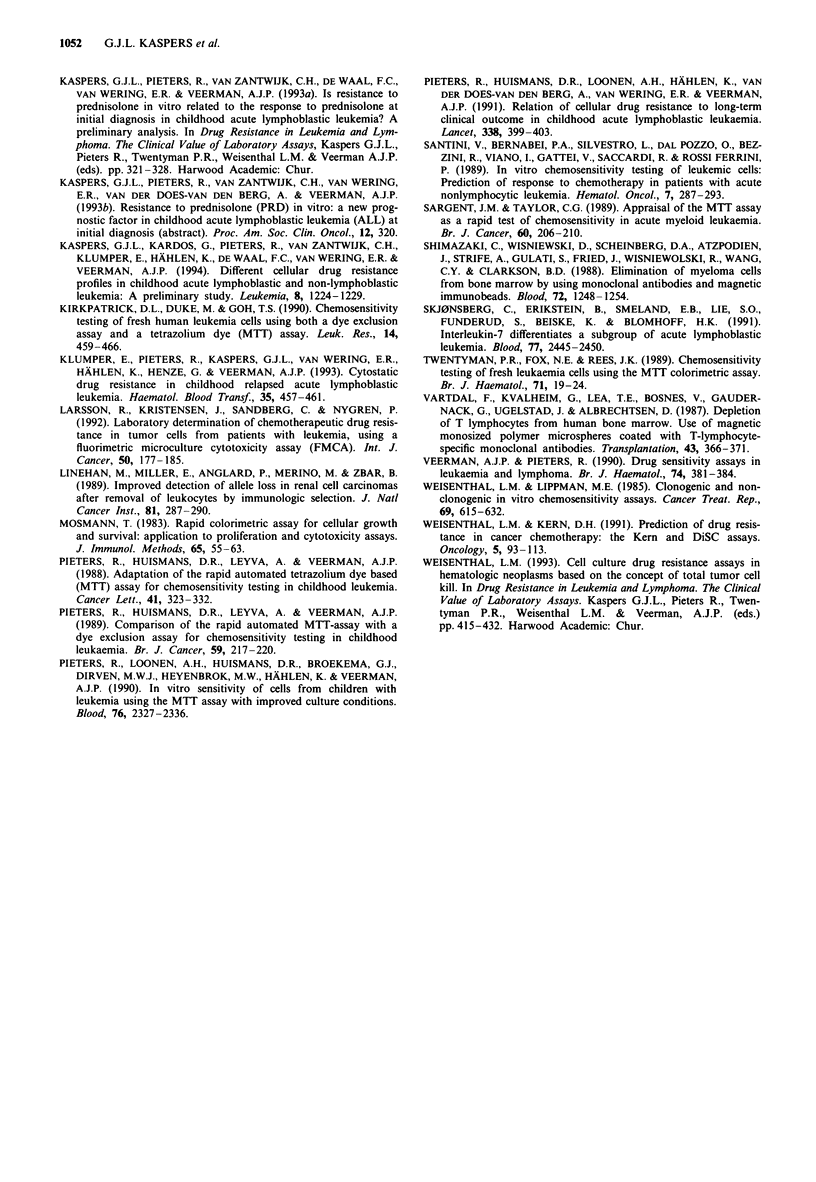# Mononuclear cells contaminating acute lymphoblastic leukaemic samples tested for cellular drug resistance using the methyl-thiazol-tetrazolium assay.

**DOI:** 10.1038/bjc.1994.446

**Published:** 1994-12

**Authors:** G. J. Kaspers, A. J. Veerman, R. Pieters, G. J. Broekema, D. R. Huismans, K. M. Kazemier, A. H. Loonen, M. A. Rottier, C. H. van Zantwijk, K. Hählen

**Affiliations:** Department of Paediatrics, Free University Hospital, Amsterdam, The Netherlands.

## Abstract

The methyl-thiazol-tetrazolium (MTT) assay is a drug resistance assay which cannot discriminate between malignant and non-malignant cells. We previously reported that samples with > or = 80% leukaemic cells at the start of culture give similar results in the MTT assay and the differential staining cytotoxicity assay, in which a discrimination between malignant and non-malignant cells can be made. However, the percentage of leukaemic cells may change during culture, which might affect the results of the MTT assay. We studied 106 untreated childhood acute lymphoblastic leukemia (ALL) samples with > or = 80% leukaemic cells at the start of culture. This percentage decreased below 80% in 28%, and below 70% in 13%, of the samples after 4 days of culture. A decrease below 70% occurred more often in case of 80-89% leukaemic cells (9/29) than in case of > or = 90% leukaemic cells at the start of culture (5/77, P = 0.0009). Samples with < 70% leukaemic cells after culture were significantly more resistant to 6 out of 13 drugs, and showed a trend towards being more resistant to two more drugs, than samples with > or = 80% leukaemic cells. No such differences were seen between samples with 70-79% and samples with > or = 80% leukaemic cells after culture. We next studied in another 30 ALL samples whether contaminating mononuclear cells could be removed by using immunoamagnetic beads. Using a beads to target cell ratio of 10:1, the percentage of leukaemic cells increased from mean 72% (s.d. 9.3%) to mean 87% (s.d. 6.7%), with an absolute increase of 2-35%. The recovery of leukaemic cells was mean 82.1% (range 56-100%, s.d. 14.0%). The procedure itself did not influence the results of the MTT assay in three samples containing only leukaemic cells. We conclude that it is important to determine the percentage of leukaemic cells at the start and at the end of the MTT assay and similar drug resistance assays. Contaminating mononuclear cells can be successfully removed from ALL samples using immunomagnetic beads. This approach may increase the number of leukaemic samples which can be evaluated for cellular drug resistance with the MTT assay or a similar cell culture drug resistance assay.


 © Macmillan Press Ltd., 1994

### Mononuclear cells contaminating acute lymphoblastic leukaemic samples tested for cellular drug resistance using the methyl-thiazol-tetrazolium assay

G.J.L. Kaspers[1], A.J.P. Veerman[1,2], R. Pieters[1], G.J. Broekema[1], D.R. Huismans[1], K.M., Kazemier[1], A.H. Loonen[1], M.A.A. Rottier[1], C.H. Van Zantwijk[1], K. Hählen[2,3] & E.R. Van Wering[2]

[1]*Department of Paediatrics, Free University Hospital, De Boelelaan 1117, 1081 HV Amsterdam;* [2]*Dutch Childhood Leukaemia Study group, Dr Van Welylaan 2, 2506 LP The Hague;* [3]*Sophia's Children Hospital, Subdivision Hemato-/Oncology, Dr Molewaterplein 60, 3015 GJ Rotterdam, The Netherlands.*

**Summary** The methyl-thiazol-tetrazolium (MTT) assay is a drug resistance assay which cannot discriminate between malignant and non-malignant cells. We previously reported that samples with $\geqslant 80\%$ leukaemic cells at the start of culture give similar results in the MTT assay and the differential staining cytotoxicity assay, in which a discrimination between malignant and non-malignant cells can be made. However, the percentage of leukaemic cells may change during culture, which might affect the results of the MTT assay. We studied 106 untreated childhood acute lymphoblastic leukaemia (ALL) samples with $\geqslant 80\%$ leukaemic cells at the start of culture. This percentage decreased below 80% in 28%, and below 70% in 13%, of the samples after 4 days of culture. A decrease below 70% occurred more often in case of 80-89% leukaemic cells (9/29) than in case of $\geqslant 90\%$ leukaemic cells at the start of culture (5/77, $P = 0.0009$). Samples with $<70\%$ leukaemic cells after culture were significantly more resistant to 6 out of 13 drugs, and showed a trend towards being more resistant to two more drugs, than samples with $\geqslant 80\%$ leukaemic cells. No such differences were seen between samples with 70-79% and samples with $\geqslant 80\%$ leukaemic cells after culture. We next studied in another 30 ALL samples whether contaminating mononuclear cells could be removed by using immunomagnetic beads. Using a beads to target cell ratio of 10:1, the percentage of leukaemic cells increased from mean 72% (s.d. 9.3%) to mean 87% (s.d. 6.7%), with an absolute increase of 2-35%. The recovery of leukaemic cells was mean 82.1% (range 56-100%, s.d. 14.0%). The procedure itself did not influence the results of the MTT assay in three samples containing only leukaemic cells. We conclude that it is important to determine the percentage of leukaemic cells at the start and at the end of the MTT assay and similar drug resistance assays. Contaminating mononuclear cells can be successfully removed from ALL samples using immunomagnetic beads. This approach may increase the number of leukaemic samples which can be evaluated for cellular drug resistance with the MTT assay or a similar cell culture drug resistance assay.

The methyl-thiazol-tetrazolium (MTT) assay is a short-term cell culture drug resistance assay. It is based on the fact that living cells, in contrast to dead cells, can reduce MTT to a coloured formazan product, which is measured spectrophotometrically. The principle was described in 1953 by Black and Speer, while Mosmann described the MTT assay in 1983 in its present semiautomated form. It is a rapid, reliable and objective assay, suited for large-scale patient studies in leukaemia and lymphoma (Veerman & Pieters, 1990). Therefore, this assay was adapted for drug resistance testing of human leukaemia samples (Hongo *et al.*, 1987; Campling *et al.*, 1988; Pieters *et al.*, 1988; Sargent & Taylor, 1989; Twentyman *et al.*, 1989; Hwang *et al.*, 1993). The results of this and other tetrazolium salt (e.g. INT)-based cellular drug resistance assays correlate well with the clinical outcome after chemotherapy (Santini *et al.*, 1989; Sargent & Taylor, 1989; Hongo *et al.*, 1990; Pieters *et al.*, 1991; Kaspers *et al.*, 1993a,b).

A disadvantage of the MTT assay, and of other assays such as the fluorometric microculture cytotoxicity assay (Larsson *et al.*, 1992) and the INT assay (Santini *et al.*, 1989), is that no discrimination can be made between malignant and non-malignant living cells. Such a discrimination can be made with the differential staining cytotoxicity (DiSC) assay (Weisenthal & Kern, 1991), but this assay is laborious and subjective (Veerman & Pieters, 1990). An alternative may be the removal of contaminating mononuclear cells from malignant samples.

We previously reported that normal peripheral blood lymphocytes are more resistant to drugs than untreated childhood acute lymphoblastic leukaemia (ALL) samples (Kaspers *et al.*, 1991). Therefore, the presence of these cells in an ALL sample will influence the MTT assay results. It has been shown that when 80% or more leukaemic cells are present in the sample at the start of the assay, the MTT assay and DiSC assay provide similar results (Pieters *et al.*, 1989; Kirkpatrick *et al.*, 1990). However, one should also consider the percentage of leukaemic cells after culture, because a decrease in this percentage may influence the assay results.

We studied in 106 untreated childhood ALL samples whether a decrease in the percentage of leukaemic cells had occurred after culture, and whether this influenced the drug resistance profile. We also investigated in another group of untreated or relapsed ALL samples whether contaminating mononuclear cells could be removed by using immunomagnetic Dynabeads. This may increase the number of samples which can be evaluated for cellular drug resistance using the MTT assay or similar drug resistance assays.

### Materials and methods

### *Cells*

Mononuclear cells were isolated from the bone marrow or peripheral blood samples by Ficoll density-gradient centrifugation (Ficol Paque; density 1.077 g $ml^{-1}$; Pharmacia, Sweden) for 15 min (room temperature, 1,000 *g*), washed twice, and resuspended in RPMI-1640 (Dutch modification, Gibco, Uxbridge, UK). The medium contained 20% fetal calf serum (Flow Laboratories, Irvine, UK) and several other supplements (Pieters *et al.*, 1990).

A total of 106 samples from children with newly diagnosed

Correspondence: G.J.L. Kaspers.
Received 5 October 1993; and in revised form 25 July 1994.

ALL with ⩾ 80% leukaemic cells were used to study changes in the percentage of leukaemic cells before and after culture and to study the possible influence of such changes on the results of cellular drug resistance testing. The percentage of leukaemic cells was determined morphologically. In case of any doubt, different technicians determined the percentage independently and immunology was used whenever possible. The samples consisted of 80 cases of precursor B-ALL [positive for terminal deoxynucleotidyl-transferase (TdT), HLA-DR and CD19), 25 cases of T-ALL (positive for TdT, cytoplasmic CD3 and CD7), and one case with unknown immunophenotype. Precursor B-ALL was subdivided into pro-B-ALL [$CD10^-$, cytoplasmic $\mu$ chain $(c\mu)^-$, $n = 6$], common ALL ($CD10^+$, $c\mu^-$, $n = 52$) and pre-B ALL ($c\mu^+$, $n = 22$). The majority of these samples had been sent to the research laboratory for paediatric haemato-onco-immunology of the Free University Hospital of Amsterdam by the laboratory of the Dutch Childhood Leukaemia Study Group for cellular drug resistance testing.

Another group of 17 samples from children with newly diagnosed ALL and 13 samples from children with relapsed ALL was used to study the removal of mononuclear cells by immunomagnetic beads and the influence of this procedure on the results of cellular drug resistance testing. Most of these samples were tested after cryopreservation.

*Cell culture drug resistance assay*

We used the colorimetric MTT assay, as described previously (Pieters *et al.*, 1990). Briefly, ALL cells were cultured in the wells of microculture plates for 4 days at a final concentration of $1.2-1.6 \times 10^6$ cells $ml^{-1}$, with or without cytotoxic agents. The 106 untreated childhood ALL samples were tested with up to 13 drugs, depending on the number of cells available: prednisolone (concentration range 0.05–1,500 $\mu g\ ml^{-1}$), dexamethasone (0.0002–6 $\mu g\ ml^{-1}$), vincristine (VCR, 0.05–50 $\mu g\ ml^{-1}$), vindesine (0.05–50 $\mu g\ ml^{-1}$), daunorubicin (0.002–2 $\mu g\ ml^{-1}$), doxorubicin (0.008–8 $\mu g\ ml^{-1}$), mitoxantrone (0.001–1 $\mu g\ ml^{-1}$), L-asparaginase (0.003–10 IU $ml^{-1}$), mercaptopurine (6MP, 15.6–500 $\mu g\ ml^{-1}$), thioguanine (1.56–50 $\mu g\ ml^{-1}$), teniposide (TEN, 0.003–8 $\mu g\ ml^{-1}$), cytarabine (ara-C, 0.002–2.5 $\mu g\ ml^{-1}$) and 4-hydroperoxyifosfamide (4-HOO-ifosfamide, 0.1–100 $\mu g\ ml^{-1}$). The samples used for the experiments with the immunomagnetic beads were usually tested with only 4–5 drugs, because of the lack of material. Most drugs were obtained from the hospital pharmacy, except that 6MP, thioguanine and doxorubicin were from Sigma (St Louis, MO, USA) and 4-HOO-ifosfamide was a gift from ASTA Pharma (Dr M. Peukert, Bielefeld, Germany). After the incubation period, MTT (Sigma) was added to all wells and the plates were again incubated for 6 h. The formed formazan crystals were dissolved with acidified (0.04 N hydrochloric acid) isopropanol, and the optical density in each well was measured with an EL-312 microplate reader (Biotek Instruments, Winooski, USA) at 562 nm. After correction for the optical density of the medium, leukaemic cell survival (LCS) was calculated by the equation: LCS = (OD treated well/mean OD control wells) × 100%. The $LC_{50}$, the concentration lethal to 50% of the cells, was calculated from the dose–response curve and used as measure for drug resistance.

*Removal of mononuclear cells using immunomagnetic Dynabeads*

The magnetic polystyrene beads (Dynabeads M-450, Dynal, Norway) were washed twice in culture medium before use. The immunophenotype of the leukaemia sample and the type of contaminating mononuclear cells as identified morphologically (mainly lymphocytes, and sometimes monocytes, erythroblasts or immature myeloid cells) determined which one of two protocols was used.

The first protocol was used in case of non-T-ALL contaminated by normal T lymphocytes, which constituted the majority (19/30) of samples. The mononuclear cells isolated after gradient centrifugation were incubated for 30 min at room temperature, with continuous gentle mixing, with the monoclonal antibody CD2 directly coated on immunomagnetic Dynabeads to remove these T lymphocytes.

The second protocol was used in the other samples, i.e. in case of non-T-ALL and T-ALL with contaminating mononuclear cells other than T lymphocytes. We did not encounter the problem of a T-ALL contaminated by normal T lymphocytes. In this second protocol, the mononuclear cells were first incubated for 30 min at 37°C with one or more of several mouse monoclonal antibodies, then washed twice with protein-buffered saline and 0.1% bovine serum albumin to remove unbound antibody, and after that incubated for 30 min at room temperature with continuous gentle mixing with Dynabeads coated with sheep anti-mouse immunoglobulin G. The choice of antibodies, used at concentrations as normally used in our laboratory for immunostaining, depended on the type of contaminating mononuclear cells: CD14 in the case of monocytes, CD15 in the case of myeloid cells (CD15 plus CD13 if immature myeloid cells were also present) and E-1 antigen in the case of erythroid cells (E-1 plus H1-antigen if immature erythroid cells were also present).

With both protocols, the tubes containing cells and Dynabeads were placed on a magnet for 2 min. This resulted in an adhesion of the target cells attached to the magnetic beads to the wall of the tubes. The suspension was aspirated and used for further studies. We used a beads to target cell (cells to be removed) ratio of 10:1. This ratio was chosen after preliminary experiments which showed that this was optimal with respect to cell loss, enrichment and costs. The effect of the Dynabeads procedure was assessed by comparing the percentage of leukaemia cells, determined morphologically, before and after the procedure.

*Statistics*

The Spearmans rank correlation test (parameter $\rho$) was used to assess the correlation between several continuous variables. The chi-squared test and Mann–Whitney *U*- or Wilcoxon test for paired and unpaired samples were used for two-tailed testing at a level of significance of 0.05. A *P*-value of $>0.05$ and $\leqslant 0.10$ was considered to indicate a trend.

### Results

*Changes in percentage of leukaemic cells*

In the group of 106 untreated childhood ALL samples, the median percentage of leukaemic cells was 93% (range 80–100%) before and 90% (range 7–99%) after the 4 days of culture. There was a significant correlation between these percentages ($\rho = 0.61$, $P < 0.000001$), as shown in Figure 1. The percentage of leukaemic cells decreased below 80% in 28%, and below 70% in 13%, of the samples after 4 days of culture (Table I). The number of cases with a decrease in the percentage of leukaemic cells below 70% was higher ($P = 0.0009$) in the case of 80–89% of leukaemic cells before the start of culture (9/29 cases, 31%), than in the case of 90% leukaemic cells at that time (5/77 cases, 6%). There was no significant relation between the occurrence of a decrease below 70% leukaemic cells and sex, age, white blood cell count at diagnosis, leukaemic cell burden, morphological FAB type, or with sample source (bone marrow or peripheral blood). This decrease below 70% occurred in two out of six pro-B, seven out of 52 common ALL, one out of 22 pre-B ALL and four out of 25 T-ALL, which was also not significantly different ($P = 0.30$).

*Percentage of leukaemic cells and drug resistance profile*

There was a significant correlation between the percentage of leukaemic blast cells after 4 days of culture and $LC_{50}$ values for nine out of 13 drugs in the group of 106 untreated ALL

samples. All of these correlations were negative, and $\rho$ values ranged from $-0.22$ to $-0.35$. Thus, lower percentages of leukaemic blast cells were associated with higher $LC_{50}$ values (i.e. a more resistant MTT assay result). No significant correlation was found for VCR, 6MP, ara-C or 4-HOO-ifosfamide. The 106 samples were divided into three groups: 14 samples with <70%, 16 samples with 70–79% and 76 samples with ≥80% leukaemic cells after culture. Table II shows median $LC_{50}$ values and ranges for all 13 drugs for these three groups. In general, samples with lower percentages of leukaemic cells showed higher $LC_{50}$ values. $LC_{50}$ values for samples with 70–79% leukaemic cells and those for samples with ≥80% leukaemic cells after culture did not differ at statistical analysis (Table II). Differences were also not significant between samples with 70–79% leukaemic cells and samples with ≥90% leukaemic cells after culture (data not shown). However, samples with <70% leukaemic cells were significantly more resistant, or showed a trend towards being more resistant to all drugs except VCR, 6MP, ara-C, TEN and 4-HOO-ifosfamide, than samples with ≥80% leukaemic cells after culture (Table II).

**Figure 1** Percentage of ALL cells before and after 4 days of culture in 106 untreated childhood ALL samples. The line shows the cut-off level (70%) below which we consider the results for samples not evaluable when the MTT assay is used.

**Table I** Change in percentage of leukaemic cells over the 4 day culture period in 106 untreated childhood ALL samples

| *Per cent at day 0* | *n* | *Per cent at day 4* | *n* |
|---|---|---|---|
| 80–84 | 12 | <70 | 4 (33%) |
| | | 70–79 | 5 (42%) |
| | | ≥80 | 3 (25%) |
| 85–89 | 17 | <70 | 5 (29%) |
| | | 70–79 | 4 (24%) |
| | | ≥80 | 8 (47%) |
| 90–94 | 42 | <70 | 5 (12%) |
| | | 70–79 | 4 (10%) |
| | | ≥80 | 33 (78%) |
| ≥95 | 35 | <70 | 0 |
| | | 70–79 | 3 (9%) |
| | | ≥80 | 32 (91%) |

There was no significant relation (Mann–Whitney $U$-test) between treatment outcome and cell viability ($P=0.67$) or MTT-specific activity (MTT dye reduction capacity, $P=0.82$). The relation between drug resistance profiles and treatment outcome will be the subject of another report. There was also no correlation between cell viability or MTT-specific activity with *in vitro* drug resistance (data not shown).

### *Removal of mononuclear cells using immunomagnetic Dynabeads*

The influence of this procedure on the results of the MTT assay was studied in three ALL samples with ≥95% leukaemic cells after isolation. Each sample was divided in two; one half was exposed to Dynabeads as described above and one half was not. For all three samples, dose–response curves for the four drugs tested were very similar for the ALL cells which had been exposed and those which had not been exposed to Dynabeads. This is illustrated in Figure 2 for one of these patients. In addition, the procedure did not change the optical density of control wells (cells, no drugs), and was not cytotoxic to the ALL cells as determined by trypan blue exclusion.

**Table II** Relation between percentage of leukaemic cells at day 4 of culture and drug resistance determined with the MTT assay in untreated childhood ALL

| | $LC_{50}$ *values in* μg ml$^{-1}$, *median (range)* | | | P-*values*[a] | | |
|---|---|---|---|---|---|---|
| *Drug* | *Group 1* <70% (n = 14) | *Group 2* 70–79% (n = 16) | *Group 3* ≥80% (n = 76) | *1 vs 2* | *1 vs 3* | *2 vs 3* |
| Prednisolone | 1500 (23.4–1500) | 2.20 (0.06–1500) | 1.03 (0.05–1500) | 0.0005 | 0.0001 | 0.60 |
| Dexamethasone | 6 (0.01–6) | 0.33 (0.01–6) | 0.09 (0.002–6) | 0.01 | 0.0001 | 0.66 |
| Vincristine | 5.55 (0.11–50) | 0.68 (0.07–28.2) | 0.71 (0.05–50) | 0.14 | 0.26 | 0.59 |
| Vindesine | 13.87 (0.65–50) | 2.64 (0.53–34.2) | 2.47 (0.05–50) | 0.13 | 0.06 | 0.97 |
| Daunorubicin | 0.34 (0.08–2) | 0.21 (0.06–0.88) | 0.12 (0.002–2) | 0.20 | 0.02 | 0.39 |
| Doxorubicin | 0.57 (0.22–8) | 0.44 (0.17–1.33) | 0.40 (0.03–8) | 0.16 | 0.08 | 0.83 |
| Mitoxantrone | 0.17 (0.04–1) | 0.10 (0.01–0.65) | 0.06 (0.001–1) | 0.15 | 0.03 | 0.49 |
| L-Asparaginase (IU ml$^{-1}$) | 4.46 (0.003–10) | 0.39 (0.002–1.88) | 0.31 (0.002–10) | 0.02 | 0.04 | 0.98 |
| Mercaptopurine | 162.5 (22.7–500) | 59.5 (15.6–500) | 101.6 (15.6–500) | 0.05 | 0.29 | 0.12 |
| Thioguanine | 13.0 (3.3–50) | 7.3 (1.8–12.2) | 6.4 (1.56–35.6) | 0.04 | 0.02 | 0.72 |
| Teniposide | 1.30 (0.19–8) | 0.32 (0.20–1.45) | 0.30 (0.06–8) | 0.34 | 0.14 | 0.48 |
| Cytarabine | 0.43 (0.03–2.5) | 0.21 (0.04–1.65) | 0.41 (0.002–2.5) | 0.41 | 0.88 | 0.16 |
| 4-HOO-Ifosfamide | 4.56 (0.27–23.56) | 3.35 (0.78–5.35) | 3.84 (0.79–20.3) | 0.18 | 0.68 | 0.14 |

[a]Mann–Whitney $U$-test for unpaired samples.

**Figure 2** Influence of the exposure of immunomagnetic beads on cellular drug resistance in an ALL sample containing only leukaemic cells before and after culture. Drug concentrations increase from A to F (Materials and methods). Dose-response curves are unchanged. **a**, -O-, Prednisolone, no beads; --O--, prednisolone, after beads; -◆-, asparaginase, no beads, --◆--, asparaginase, after beads. **b**, -O-, Daunorubicin, no beads; --O--, daunorubicin, after beads; -◆-, vincristine, no beads; --◆--, vincristine, after beads.

The removal of mononuclear cells contaminating leukaemic samples was tried in an additional 30 ALL samples, and the results are shown in Table III. The percentage of leukaemic cells increased from mean 72.0% (s.d. 9.3%) before to mean 87.0% (s.d. 6.7%) leukaemic cells after the Dynabeads procedure. In 17 (77%) out of 22 samples with <80% leukaemic cells, this percentage increased to ⩾80% after removal of mononuclear cells. The recovery of leukaemic cells was mean 82.1% (s.d. 14.0%, range 56-100%). There was no correlation between the recovery of leukaemic cells with the total cell number exposed to Dynabeads ($\rho = 0.06$, $P = 0.79$) or with the percentage of leukaemic cells in the sample before the exposure to Dynabeads ($\rho = 0.07$, $P = 0.77$). The percentage of trypan blue-positive dead cells decreased from mean 12.5% (s.d. 15.1%) before to mean 7.4% (s.d. 9.8%) after the Dynabeads exposure.

Whether the removal of mononuclear cells from ALL samples actually resulted in a change in drug resistance profile-towards a more sensitive profile-was tested by a paired comparison in one sample. The results showed this indeed to be the case (Figure 3). This was later confirmed in two additional samples.

In 24 out of 30 samples the percentage of leukaemic cells was ⩾80% at the start of culture, that is after the Dynabeads procedure. Two (9%) out of these 24 samples, both from patients with relapsed pre-B-ALL, showed a decrease below 70% (62% and 49%) leukaemic cells after culture. This frequency of 9% is similar to that of 13% in samples not exposed to Dynabeads, as mentioned above. The Dynabeads procedure did not have a significant adverse effect on the control cell viability in drug-free wells after 4 days of culture (mean 54.8%, s.d. 18.4%), as compared with the control cell viability in the 106 untreated ALL samples not exposed to Dynabeads (mean 63.0%, s.d. 28.9%).

**Table III** Results of removal of contaminating mononuclear cells from 30 ALL samples using immunomagnetic beads

| | *After beads* |
|---|---|
| Increase in percentage of leukaemic cells | |
| Mean ± s.d. | 15.3 ± 8.4% |
| Median (range) | 16% (2-35%) |
| Recovery of leukaemic cells (%) | |
| Mean ± s.d. | 82.1 ± 14.0% |
| Median (range) | 80% (56-100%) |
| Change in percentage of trypan blue-positive cells | |
| Mean ± s.d. | −3.7 ± 6.7 |
| Median (range) | 0% (−13-+8%) |
| Cell survival after 4 days of culture (%) | |
| Mean ± s.d. | 54.8 ± 18.4% |
| Median (range) | 56% (20-100%) |
| Percentage of leukaemic cells after 4 days of culture | |
| Mean ± s.d. | 74.5 ± 27.5% |
| Median (range) | 86% (10-98%) |

**Figure 3** Influence of the removal of mononuclear cells contaminating an ALL sample, using immunomagnetic beads, on cellular drug resistance. Drug concentrations increase from A to F (Materials and methods). The part of the ALL sample not exposed to beads contained 61% leukaemic cells after culture, and showed a more resistant profile than the part of the ALL sample which was exposed to beads, and which contained 96% leukaemic cells after culture. For key, see legend to Figure 2.

### Discussion

Weisenthal (1993) reported that a decrease in the percentage of leukaemic cells during 4 days of culture may occur in ALL samples. In this study we confirm this finding for untreated childhood ALL samples. The percentage of leukaemic cells was determined by morphology, a subjective method. However, in case of any doubt, several technicians determined the percentage independently, and immunology was used whenever possible. The percentages are thus the result of a careful assessment. In 106 samples containing ⩾80% leukaemic cells at the start of culture, 28% of the samples contained <80% and 13% of the samples contained <70% leukaemic cells after culture in the control wells. These latter samples were significantly more resistant to cytotoxic agents than samples with ⩾80% leukaemic cells after culture. A decrease below 70% leukemic cells, or the presence of >30% mononuclear non-malignant cells, thus influences the results of the MTT assay for untreated ALL samples. This can be explained by the fact that contaminating mononuclear cells are more drug resistant than these ALL cells, and by the inability of the MTT assay to discriminate between malignant and non-malignant cells (Kaspers *et al.*, 1991). An alternative explanation is that intrinsically resistant ALL samples tend to show a decrease in the percentage of ALL cells. However, we did not find a significant relation between leukaemia features, such as immunophenotype, and a decrease in percentage of leukaemic cells below 70%. Moreover, there was no relation between leukaemic cell viability and *in vitro* drug resistance or clinical outcome.

For ALL samples with ⩾80% leukaemic cells at the start of culture, the results of the MTT assay and DiSC assay are similar (Pieters *et al.*, 1989; Kirkpatrick *et al.*, 1990). The MTT assay can therefore be used to determine the drug resistance profile of untreated ALL samples with ⩾80% leukaemic cells present at the start of culture, and the results should not be considered evaluable in the case of the presence of <70% leukaemic cells after culture. These cautionary notes also apply for other cell culture drug resistance assays in which no distinction can be made between malignant and non-malignant cells. The cut-off points of 80% and 70% might also be appropriate for relapsed ALL samples and acute non-lymphoblastic leukaemia samples. These samples are generally not more drug sensitive than untreated ALL samples; in fact, they are significantly more resistant to certain drugs (Kaspers *et al.*, 1994; Klumper *et al.*, 1993). Therefore, contamination with mononuclear cells is less likely to result in a false, over-resistant drug profile than in the case of untreated ALL samples, assuming that similar percentages of leukaemic cells are present in these leukaemia samples.

One alternative way to determine the drug resistance profile of samples with <80% ALL cells is the DiSC assay, which distinguishes drug effects on malignant and non-malignant cells (Weisenthal & Lippman, 1985). The procedure of the DiSC assay is similar to that of the MTT assay until the moment of adding MTT to the wells. In fact, it is possible to first determine the percentage of leukaemic cells after culture, and only then to decide to use the MTT assay, in the case of ⩾70% leukaemic cells, or the DiSC assay, in the case of <70% leukaemic cells. However, the DiSC assay is laborious, subjective and requires skilled technicians (Pieters, 1989; Weisenthal & Kern, 1991). Alternatively, one may decide to use shorter drug incubation periods. However, in that case higher drug concentrations are needed to give significant cell kill in ALL samples (Pieters *et al.*, 1988). Such concentrations would far exceed clinically achievable peak plasma levels.

Another alternative would be to remove contaminating non-malignant cells to enable the use of the MTT assay for samples with initially <80% leukaemic cells or even for samples with 80–90% leukaemic cells. A marked part of these latter samples will show a decrease below 70% leukaemic cells after culture. We investigated the removal of mononuclear cells by immunomagnetic Dynabeads. These magnetic beads have been used for purging (Shimazaki *et al.*, 1988; Gruhn *et al.*, 1991), T-cell depletion of bone marrow grafts (Vartdal *et al.*, 1987), removal of leucocytes from renal carincomas prior to chromosome studies (Linehan *et al.*, 1989) and purifying ALL samples by removing T-lymphocytes and monocytes for proliferation studies (Skjønsberg *et al.*, 1991). In the present study it appeared to be possible to increase the percentage of leukaemic cells to ⩾80% in 17 out of 22 ALL samples by Dynabeads. The mean increase of the percentage of leukaemic cells after the Dynabeads procedure was 15%. The mean recovery of viable leukaemic cells was ±80%, i.e. 20% of the cells were lost. The procedure with Dynabeads did not alter the drug resistance profile of the leukaemic cells and had no adverse effect on the success rate of the MTT assay as compared with normally processed samples. It even diminished the number of dead cells in the sample. In our laboratory, we now use Dynabeads in 15–20% of all samples received for drug resistance testing. Criteria to use beads are the presence of at least $5 \times 10^6$ leukaemic cells and the presence of ⩾10% potentially removable contaminating cells. In our experience, the use of beads increases the percentage of samples with evaluable MTT assay results from 70 to 80%. These criteria and the effect on the evaluability rate of the MTT assay may differ between laboratories, and for instance largely depend on the type of malignant samples tested.

We conclude that a decrease in the percentage of leukaemic cells can occur during the culture of untreated childhood ALL samples. This decrease should be monitored if a sample is studied using the MTT assay or a similar assay, because the results are significantly influenced in case of a decrease below 70% leukaemic cells after culture. Removal of contaminating mononuclear cells is feasible using immunomagnetic Dynabeads, without an adverse effect on the leukaemic cells itself. Such a removal will increase the number of leukaemia samples which can be evaluated for cellular drug resistance using the MTT assay. This study does not answer the question whether this approach provides more reliable results for individual cases than the DiSC assay.

Some of the samples included in this study were tested in connection with our studies on cellular drug resistance in childhood leukaemia in cooperation with the German ALL-BFM Relapse Group (Professor G. Henze), the German CoALL Group (Professor G.E. Janka-Schaub), and the Dutch Childhood Leukemia Study Group (DCLSG, Dr A. Van Der Does-Van Den Berg, Dr E.R. Van Wering, Professor W.A. Kamps). Board members of the DCLSG are H. Van Den Berg, M.V.A. Bruin, J.P.M. Bökkerink, P.J. Van Dijken, K. Hählen, W.A. Kamps, F.A.E. Nabben, A. Postma, J.A. Rammeloo, I.M. Risseeuw-Appel, A.Y.N. Schouten-Van Meeteren, G.A.M. De Vaan, E. Th. Van 't Veer-Korthof, A.J.P. Veerman, M. Van Weel-Sipman and R.S. Weening.

This study was supported by the Dutch Cancer Society (IKA 89-06) and by the project VONK (VU Onderzoek Naar Kinderkanker). Computer equipment was provided by Olivetti Nederland BV.

### References

BLACK, M.M. & SPEER, F.D. (1953). Effects of cancer chemotherapeutic agents on dehydrogenase activity of human cancer tissue in vitro. *Am. J. Clin. Pathol.*, **23**, 218–227.

CAMPLING, B.G., PYM, J., GALBRAITH, P.R. & COLE, S.P.C. (1988). Use of the MTT assay for rapid determination of chemosensitivity of human leukemic blast cells. *Leuk. Res.*, **14**, 823–831.

GRUHN, B., HÄFER, R., MÜLLER, A., ANDRÄ, W., DANAN, H. & ZINTL, F. (1991). Model experiments for immunomagnetic elimination of leukemic cells from human bone marrow. Presentation of a novel magnetic separation system. *Immunobiology*, **183**, 374–385.

HONGO, T., FUJII, Y., MIZUNO, Y., HARAGUCHI, S. & YOSHIDA, T.O. (1987). Anticancer drug sensitivity test using the short-term microplate culture and MTT dye reduction assay. *Jpn J. Cancer Chemother.*, **14**, 472–478.

HONGO, T., FUJII, Y. & IGARASHI, Y. (1990). An in vitro chemosensitivity test for the screening of anticancer drugs in childhood leukemia. *Cancer*, **65**, 1263–1272.

HWANG, W.-S., CHEN, L.-M., HUANG, S.-H., WANG, C.-C. & TSENG, M.T. (1993). Prediction of chemotherapy response in human leukemia using in vitro chemosensitivity test. *Leuk. Res.*, **17**, 685–688.

KASPERS, G.J.L., PIETERS, R., VAN ZANTWIJK, C.H., DE LAAT, P.A.J.M., DE WAAL, F.C., VAN WERING, E.R. & VEERMAN, A.J.P. (1991). In vitro drug sensitivity of normal peripheral blood lymphocytes and childhood leukaemic cells from bone marrow and peripheral blood. *Br. J. Cancer*, **64**, 469–474.

KASPERS, G.J.L., PIETERS, R., VAN ZANTWIJK, C.H., DE WAAL, F.C., VAN WERING, E.R. & VEERMAN, A.J.P. (1993*a*). Is resistance to prednisolone in vitro related to the response to prednisolone at initial diagnosis in childhood acute lymphoblastic leukemia? A preliminary analysis. In *Drug Resistance in Leukemia and Lymphoma. The Clinical Value of Laboratory Assays*, Kaspers G.J.L., Pieters R., Twentyman P.R., Weisenthal L.M. & Veerman A.J.P. (eds). pp. 321–328. Harwood Academic: Chur.

KASPERS, G.J.L., PIETERS, R., VAN ZANTWIJK, C.H., VAN WERING, E.R., VAN DER DOES-VAN DEN BERG, A. & VEERMAN, A.J.P. (1993*b*). Resistance to prednisolone (PRD) in vitro: a new prognostic factor in childhood acute lymphoblastic leukemia (ALL) at initial diagnosis (abstract). *Proc. Am. Soc. Clin. Oncol.*, **12**, 320.

KASPERS, G.J.L., KARDOS, G., PIETERS, R., VAN ZANTWIJK, C.H., KLUMPER, E., HÄHLEN, K., DE WAAL, F.C., VAN WERING, E.R. & VEERMAN, A.J.P. (1994). Different cellular drug resistance profiles in childhood acute lymphoblastic and non-lymphoblastic leukemia: A preliminary study. *Leukemia*, **8**, 1224–1229.

KIRKPATRICK, D.L., DUKE, M. & GOH, T.S. (1990). Chemosensitivity testing of fresh human leukemia cells using both a dye exclusion assay and a tetrazolium dye (MTT) assay. *Leuk. Res.*, **14**, 459–466.

KLUMPER, E., PIETERS, R., KASPERS, G.J.L., VAN WERING, E.R., HÄHLEN, K., HENZE, G. & VEERMAN, A.J.P. (1993). Cytostatic drug resistance in childhood relapsed acute lymphoblastic leukemia. *Haematol. Blood Transf.*, **35**, 457–461.

LARSSON, R., KRISTENSEN, J., SANDBERG, C. & NYGREN, P. (1992). Laboratory determination of chemotherapeutic drug resistance in tumor cells from patients with leukemia, using a fluorimetric microculture cytotoxicity assay (FMCA). *Int. J. Cancer*, **50**, 177–185.

LINEHAN, M., MILLER, E., ANGLARD, P., MERINO, M. & ZBAR, B. (1989). Improved detection of allele loss in renal cell carcinomas after removal of leukocytes by immunologic selection. *J. Natl Cancer Inst.*, **81**, 287–290.

MOSMANN, T. (1983). Rapid colorimetric assay for cellular growth and survival: application to proliferation and cytotoxicity assays. *J. Immunol. Methods*, **65**, 55–63.

PIETERS, R., HUISMANS, D.R., LEYVA, A. & VEERMAN, A.J.P. (1988). Adaptation of the rapid automated tetrazolium dye based (MTT) assay for chemosensitivity testing in childhood leukemia. *Cancer Lett.*, **41**, 323–332.

PIETERS, R., HUISMANS, D.R., LEYVA, A. & VEERMAN, A.J.P. (1989). Comparison of the rapid automated MTT-assay with a dye exclusion assay for chemosensitivity testing in childhood leukaemia. *Br. J. Cancer*, **59**, 217–220.

PIETERS, R., LOONEN, A.H., HUISMANS, D.R., BROEKEMA, G.J., DIRVEN, M.W.J., HEYENBROK, M.W., HÄHLEN, K. & VEERMAN, A.J.P. (1990). In vitro sensitivity of cells from children with leukemia using the MTT assay with improved culture conditions. *Blood*, **76**, 2327–2336.

PIETERS, R., HUISMANS, D.R., LOONEN, A.H., HÄHLEN, K., VAN DER DOES-VAN DEN BERG, A., VAN WERING, E.R. & VEERMAN, A.J.P. (1991). Relation of cellular drug resistance to long-term clinical outcome in childhood acute lymphoblastic leukaemia. *Lancet*, **338**, 399–403.

SANTINI, V., BERNABEI, P.A., SILVESTRO, L., DAL POZZO, O., BEZZINI, R., VIANO, I., GATTEI, V., SACCARDI, R. & ROSSI FERRINI, P. (1989). In vitro chemosensitivity testing of leukemic cells: Prediction of response to chemotherapy in patients with acute nonlymphocytic leukemia. *Hematol. Oncol.*, **7**, 287–293.

SARGENT, J.M. & TAYLOR, C.G. (1989). Appraisal of the MTT assay as a rapid test of chemosensitivity in acute myeloid leukaemia. *Br. J. Cancer*, **60**, 206–210.

SHIMAZAKI, C., WISNIEWSKI, D., SCHEINBERG, D.A., ATZPODIEN, J., STRIFE, A., GULATI, S., FRIED, J., WISNIEWOLSKI, R., WANG, C.Y. & CLARKSON, B.D. (1988). Elimination of myeloma cells from bone marrow by using monoclonal antibodies and magnetic immunobeads. *Blood*, **72**, 1248–1254.

SKJØNSBERG, C., ERIKSTEIN, B., SMELAND, E.B., LIE, S.O., FUNDERUD, S., BEISKE, K. & BLOMHOFF, H.K. (1991). Interleukin-7 differentiates a subgroup of acute lymphoblastic leukemia. *Blood*, **77**, 2445–2450.

TWENTYMAN, P.R., FOX, N.E. & REES, J.K. (1989). Chemosensitivity testing of fresh leukaemia cells using the MTT colorimetric assay. *Br. J. Haematol.*, **71**, 19–24.

VARTDAL, F., KVALHEIM, G., LEA, T.E., BOSNES, V., GAUDERNACK, G., UGELSTAD, J. & ALBRECHTSEN, D. (1987). Depletion of T lymphocytes from human bone marrow. Use of magnetic monosized polymer microspheres coated with T-lymphocyte-specific monoclonal antibodies. *Transplantation*, **43**, 366–371.

VEERMAN, A.J.P. & PIETERS, R. (1990). Drug sensitivity assays in leukaemia and lymphoma. *Br. J. Haematol.*, **74**, 381–384.

WEISENTHAL, L.M. & LIPPMAN, M.E. (1985). Clonogenic and nonclonogenic in vitro chemosensitivity assays. *Cancer Treat. Rep.*, **69**, 615–632.

WEISENTHAL, L.M. & KERN, D.H. (1991). Prediction of drug resistance in cancer chemotherapy: the Kern and DiSC assays. *Oncology*, **5**, 93–113.

WEISENTHAL, L.M. (1993). Cell culture drug resistance assays in hematologic neoplasms based on the concept of total tumor cell kill. In *Drug Resistance in Leukemia and Lymphoma. The Clinical Value of Laboratory Assays*. Kaspers G.J.L., Pieters R., Twentyman P.R., Weisenthal L.M. & Veerman, A.J.P. (eds.) pp. 415–432. Harwood Academic: Chur.